# A simple selection-free method for detecting disseminated tumor cells (DTCs) in murine bone marrow

**DOI:** 10.18632/oncotarget.12000

**Published:** 2016-09-13

**Authors:** Kenneth C. Valkenburg, Sarah R. Amend, James E. Verdone, Emma E. van der Toom, James R. Hernandez, Michael A. Gorin, Kenneth J. Pienta

**Affiliations:** ^1^ The James Buchanan Brady Urological Institute and Department of Urology, Johns Hopkins University School of Medicine, Baltimore, MD 21287, USA; ^2^ Department of Oncology, Johns Hopkins University School of Medicine, Baltimore, MD 21287, USA; ^3^ Department of Pharmacology and Molecular Sciences, Johns Hopkins University School of Medicine, Baltimore, MD 21287, USA; ^4^ Department of Chemical and Biomolecular Engineering, Johns Hopkins University, Baltimore, MD 21218, USA

**Keywords:** disseminated tumor cells, mouse models, bone marrow, bone metastasis, cancer

## Abstract

Bone metastasis is a lethal and incurable disease. It is the result of the dissemination of cancer cells to the bone marrow. Due to the difficulty in sampling and detection, few techniques exist to efficiently and consistently detect and quantify disseminated tumor cells (DTCs) in the bone marrow of cancer patients. Because mouse models represent a crucial tool with which to study cancer metastasis, we developed a novel method for the simple selection-free detection and quantification of bone marrow DTCs in mice. We have used this protocol to detect human and murine DTCs in xenograft, syngeneic, and genetically engineered mouse models. We are able to detect and quantify bone marrow DTCs in mice that do not have overt bone metastasis. This protocol is amenable not only for detection and quantification purposes but also to study the expression of markers of numerous biological processes or tissue-specificity.

## INTRODUCTION

Bone metastasis leads to approximately 280,000–350,000 cancer-related deaths in the United States each year [1, 2]. Prostate, breast, and lung cancers account for about 70% of these cases; kidney cancer also frequently metastasizes to bone [1, 3]. Bone metastasis occurs through a complex cascade of events that ultimately results in circulating tumor cells (CTCs) from the blood extravasating and invading the bone marrow – these cells within the bone marrow are referred to as disseminated tumor cells (DTCs) [4]. DTCs occupy the hematopoietic stem cell niche within the bone marrow [5], often remaining dormant for years before becoming reactivated, leading to cancer recurrence [6]. The factors and cellular processes required for the induction of dormancy and subsequent tumor cell reactivation remain largely undefined [6]. Beyond single DTCs, death from bone metastasis may also be due to dissemination of disease via metastatic re-seeding from other secondary sites [7, 8]. In addition, metastatic sites may re-seed primary tumors in a multi-directional fashion [8]. These important and unanswered biological questions about bone marrow DTCs and the metastatic process underscore the need for efficient and consistent methods for DTC detection in model systems.

Detection of DTCs in bone marrow aspirates of cancer patients is indicative of worse prognosis. For example, the detection of DTCs in breast cancer patients has been associated with lymph node involvement and a higher risk of relapse [9, 10]. Bone marrow DTCs have been found in 36% of metastatic breast cancer patients, and this was associated with increased metastasis and reduced overall survival [11]. In prostate cancer patients, bone marrow DTCs have been found in 72% of patients prior to radical prostatectomy, and detection of DTCs in patients with no evidence of disease was a predictor of biochemical recurrence [12]. Little is known about the prognosis of patients with other cancer types with bone marrow DTCs.

Mouse models have been employed for decades to better understand cancer metastasis [13–19]. Few mouse models fully recapitulate the entire process of tumor metastasis, so there are multiple types of models available for the study of various aspects of metastasis. These include xenograft models (human cells injected into immunodeficient mice), syngeneic models (murine cells injected into immune competent mice with the same genetic background), and genetically engineered models (including transgenic and knockout mouse models) [20]. Mouse models provide a way to validate genetic pathways in cancer and find novel therapies to treat cancer [21].

There are currently no standardized methods for the detection of bone marrow DTCs in the clinical setting. Similarly, despite the essential role of experimental mouse models to study DTC biology, few strategies exist for the consistent detection and quantification of DTCs in mouse bone marrow. Owing to the small number of DTCs relative to the large number of normal bone marrow cells (BMCs), which are largely composed of white blood cells, the process of detecting DTCs in bone marrow is difficult. The number of BMCs in one mouse leg (approximately 15–25 million) is orders of magnitude higher than the number of DTCs that may be found there. We have developed a simple selection-free method for the detection and quantification of bone marrow DTCs in mice. Importantly, this method also allows for the study of various tissue-specific and biologically interesting molecular markers.

## RESULTS

Commonly used methods for detecting CTCs in blood employ either positive or negative selection to enrich for rare tumor cells in an exceedingly large BMC population. Many of the more common assays employ a positive selection strategy, utilizing ferromagnetic particle bound anti-EpCAM antibodies to select epithelial CTCs, based on the assumption that carcinoma cells typically express epithelial markers while white blood cells (WBCs) in the blood do not [22]. One potential issue with using EpCAM to select CTCs or DTCs is that tumor cells are known to decrease this and other epithelial markers during the process of epithelial-to-mesenchymal transition [23]. Negative selection strategies are also frequently employed to enrich for tumor cells, typically using the WBC marker CD45 to deplete white blood cells from a sample. This method may result in loss of cancer cells from the sample, as some CTCs are inadvertently removed during the depletion step.

**Figure 1:**
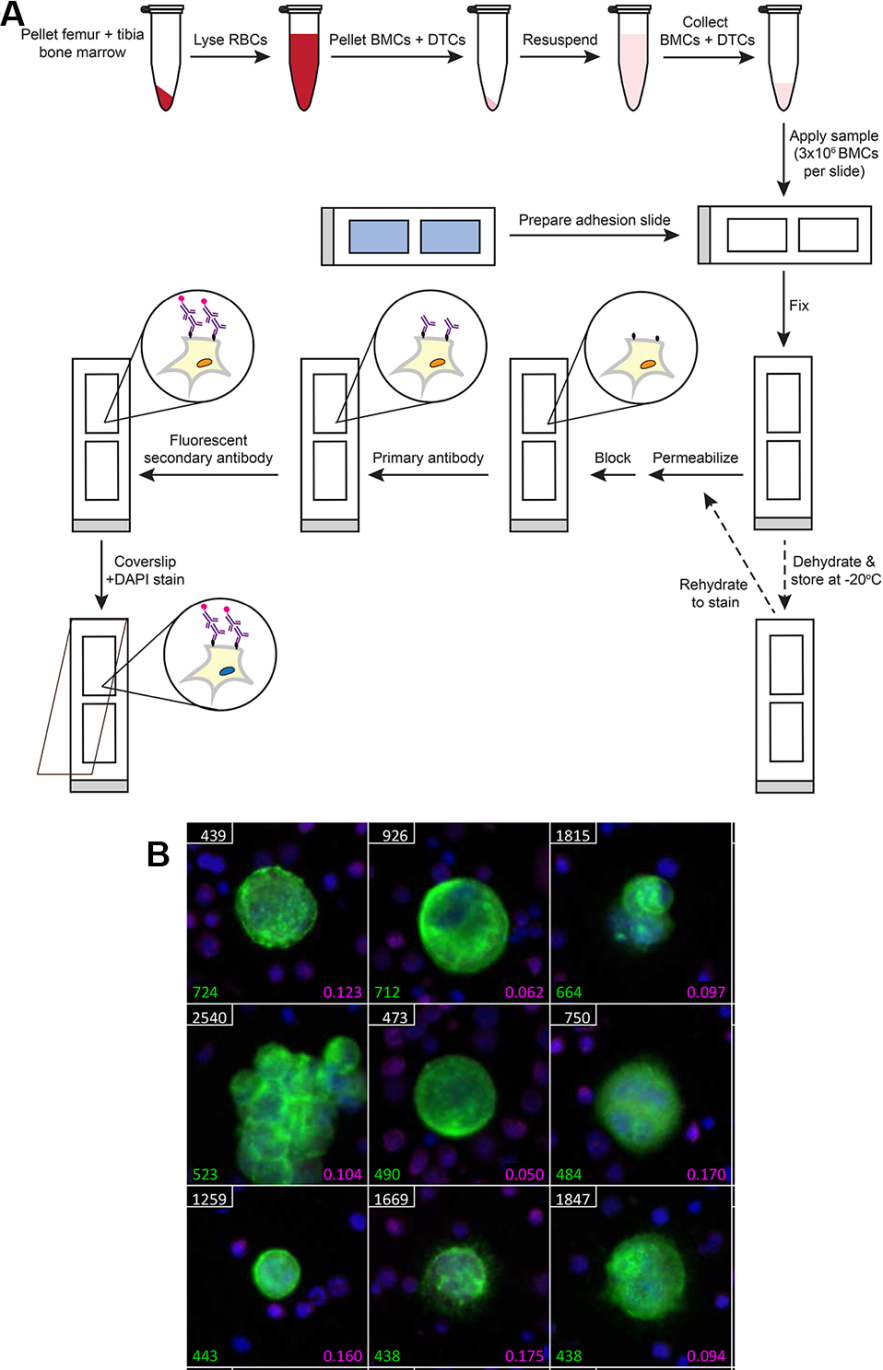
Protocol for murine bone marrow DTC detection (**A**) Diagram of the protocol. RBC = red blood cell; BMCs = bone marrow cells; and DTCs = disseminated tumor cells. (**B**) Representative gallery of DTCs generated after scanning a slide containing murine bone marrow from a cancer-injected mouse. Cells were segregated based on nuclear DAPI staining. Green = HLA-GFP; magenta = CD45-AF647; and blue = DAPI staining. White numbers represent cell position on the slide, green numbers represent HLA-GFP fluorescent intensity of candidate DTCs, and magenta numbers represent CD45-AF647 fluorescent intensity of BMCs.

We have developed a method of bone marrow DTC detection and quantification that avoids any potential bias and has high tumor cell recovery. Our protocol relies on the ability to remove bone marrow from murine bones, which is done via centrifugation of the marrow out of the bone into a microcentrifuge tube [24]. Red blood cells are lysed, and the marrow solution washed in PBS. A re-suspended portion of the sample containing 3 million BMCs is applied to proprietary adhesion slides. The sample is then fixed, permeabilized, and stained according to standard immunofluorescence protocols. Slides may also be dehydrated and frozen for staining at a later date, allowing for an entire bone marrow sample to be analyzed using this protocol (Figure 1A). To identify human DTCs in xenograft studies, slides are stained with an antibody against human leukocyte antigen (HLA), a human-specific marker. Slides are also stained with DAPI to identify nuclei and an antibody against the WBC marker CD45 to identify BMCs (note that CD45 stains approximately 65% of BMCs). Stained slides are then scanned using an automated microscope, and accompanying software generates galleries of candidate DTCs based on fluorescent intensity (Figure 1B). The resulting image galleries are manually reviewed, and true DTCs (DAPI+HLA+CD45−) are enumerated.

**Figure 2:**
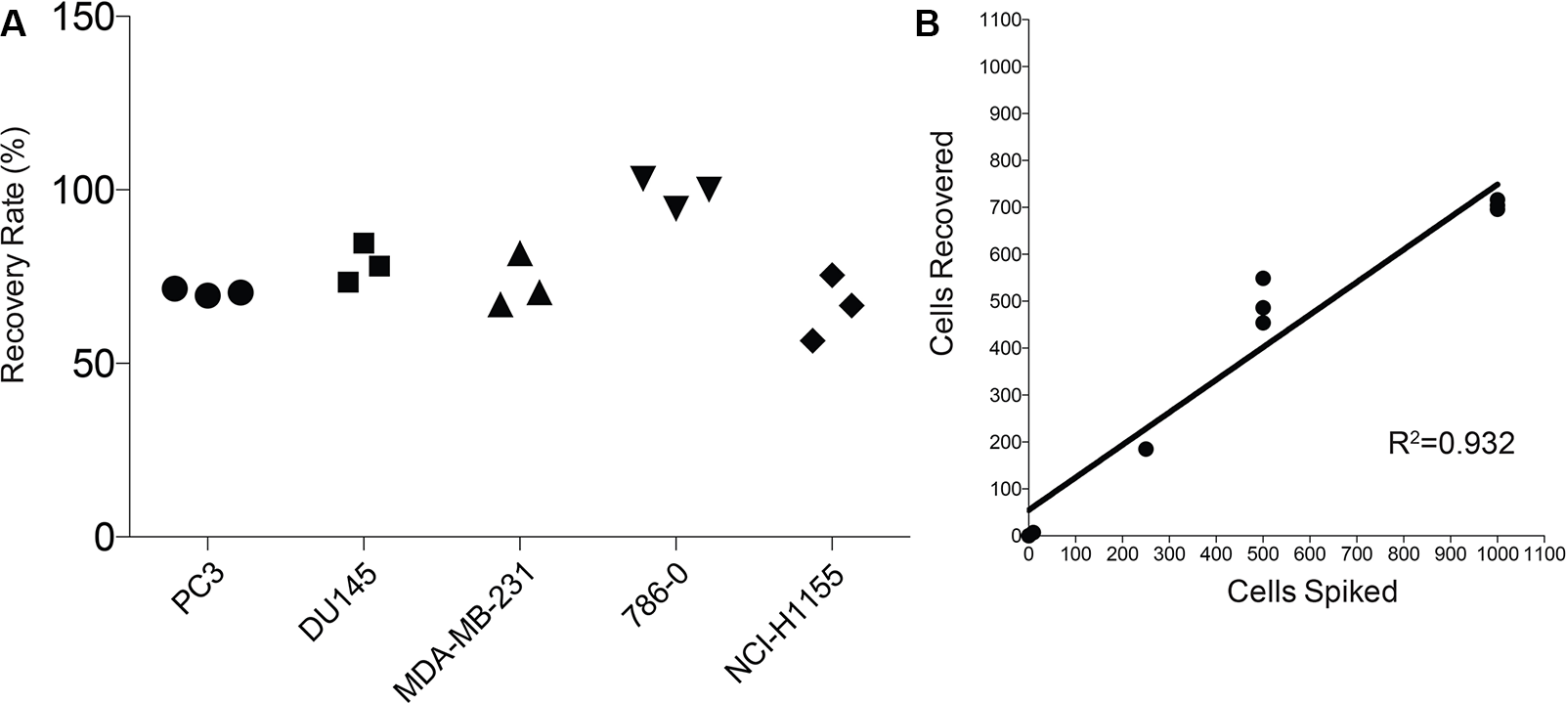
Efficiency of the protocol using cell spiking experiments (**A**) Recovery of five cancer cell lines (PC3, DU145, MDA-MB-231, 786-0, and NCI-H1155) after spiking 1000 cells into 1.5 million murine bone marrow cells (*n* = 3/cell line). (**B**) Recovery of PC3 cells after spiking 10, 250, 500, and 1000 cells into 1.5 million murine bone marrow cells (*n* = 3).

We first conducted cell-spiking experiments to determine the recovery rate of our novel method. A known number of cancer cells were spiked into mouse bone marrow, which was then subjected to the staining protocol, scanned, and quantified. The protocol consistently recovered at least 70% of spiked cancer cells across the five bone-homing cancer cell lines tested (Figure 2A). To test our ability to linearly detect cancer cells, we spiked 10, 250, 500, and 1000 cells into bone marrow and consistently detected 70–90% of spiked cells (Figure 2B).

**Figure 3:**
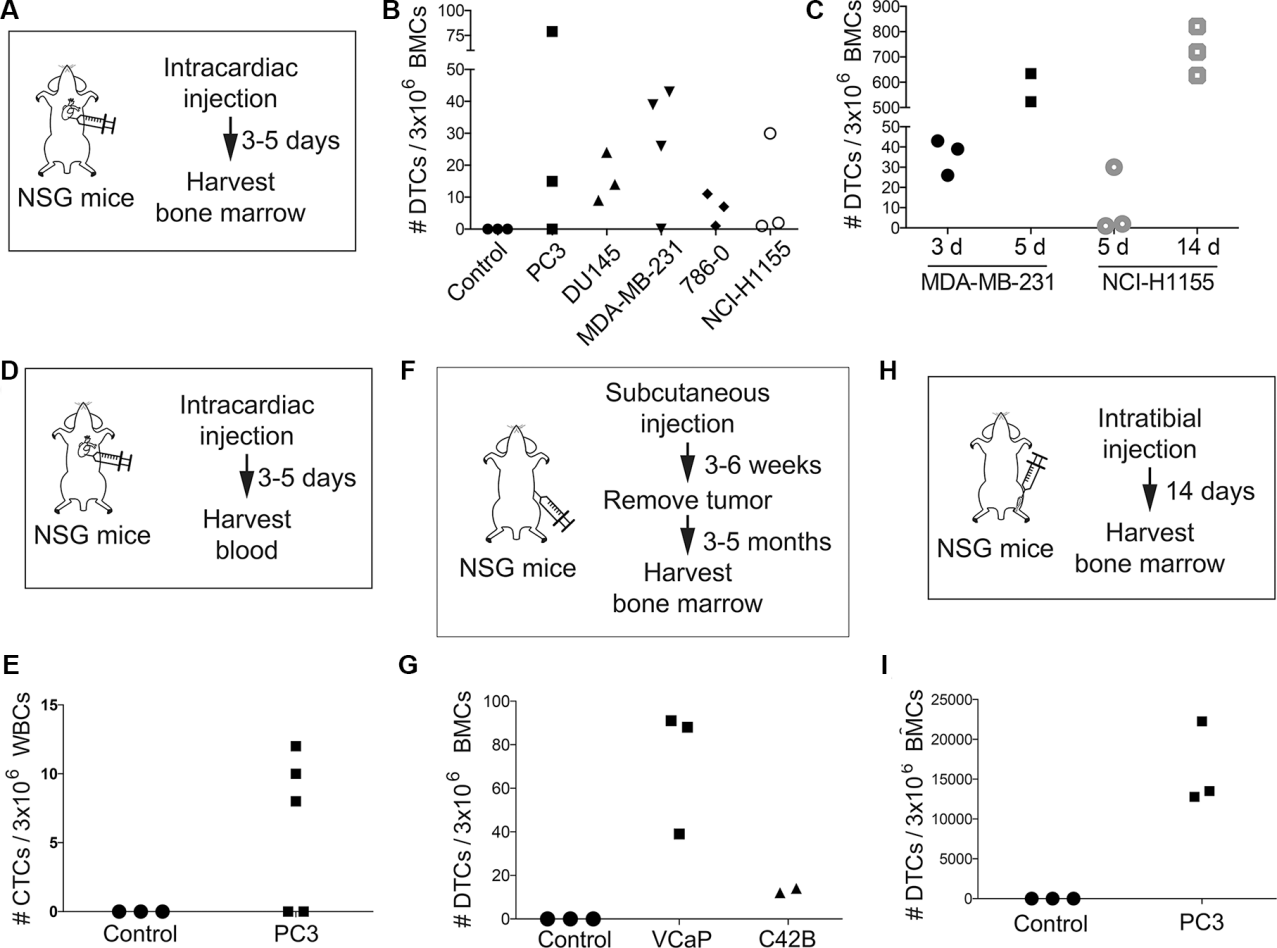
Consistent detection, quantification, and visualization of bone marrow DTCs Diagram of (**A**, **D**) intracardiac, (**F**) subcutaneous, and (**H**) intratibial injection strategies in NSG mice for bone marrow (A, F, H) or blood (**D**) collection. (**B**) Quantification of DTCs from bone marrow of NSG mice following intracardiac injection of five cancer cell lines. Note: bone marrow was harvested 5 days post-injection for each cell line except for the MDA-MB-231 line, which was harvested at 3 days post-injection. (**C**) Quantification of DTCs from bone marrow of NSG mice at various time points following intracardiac injection of MDA-MB-231 (3 days and 5 days) or NCI-H1155 cells (5 days and 14 days). (**E**) Quantification of CTCs from blood of NSG mice following intracardiac injection of PC3 cells. (**G**) Quantification of DTCs from bone marrow of NSG mice following subcutaneous injection of VCaP and C42B cells. (**I**) Quantification of DTCs from bone marrow of NSG mice following intratibial injection of PC3 cells.

Mouse xenograft (human in mouse) models of bone metastasis typically rely on some combination of intracardiac, intratibial, and/or subcutaneous injections of tumor cells into immunodeficient mice. We injected immunodeficient non-obese diabetic *SCID* interleukin 2 receptor gamma null (NSG) mice with cancer cells that are known to metastasize to bone (Supplementary Table S1). First, we performed intracardiac (IC) injections of 1 million cells and sacrificed mice three to five days later (Figure 3A). We typically sampled 3 million BMCs per sample per slide, and therefore report the DTC density as “DTCs per 3 million BMCs.” We found an average of 19 bone marrow DTCs per 3 million BMCs and in some cases found as few as 1 cell, indicating the sensitivity of the protocol (Figure 3B). No DTCs were found in non-injected controls (Figure 3B). MDA-MB-231 cells were particularly aggressive, and had approximately as many DTCs at three days post-injection as other cell lines had at five (Figure 3B). We could also detect an increase in DTCs over time. In mice bearing NCI-H1155 tumors, we observed an increase of DTCs from day 5 (average of 11 DTCs) to day 14 (average of 723 DTCs) post-inoculation (Figure 3C). Similarly, in mice bearing MDA-MB-231 tumors, we observed an increase of DTCs from day 3 (average of 27 DTCs) to day 5 (average of 579 DTCs) post-inoculation (Figure 3C). In addition to bone marrow DTC detection, we also detected CTCs from blood of IC inoculated mice (Figure 3D), with an average of 3 CTCs detected in PC3-injected mice 5 days post-injection (Figure 3E).

We next performed subcutaneous (SQ) injections using 1 million prostate cancer cells (C42B and VCaP; see Supplementary Table S1). After SQ tumors had formed, we removed the tumors and allowed the mice to age to different time-points months after injection but prior to detection of clinical metastasis (Figure 3F). We consistently detected bone marrow DTCs from these mice (Figure 3G). Relative to other inoculation models, we found fewer DTCs much longer after injection. This is likely due to the fact that SQ tumor cells go through the entire process of metastasis in order to get to the bone, whereas IC- or IT-injected cells have more direct access to the bone tissue. We also performed intratibial (IT) injections using 200,000 prostate-derived PC3 cells (Figure 3H). Two weeks after injection, we found thousands of bone marrow DTCs per 3 million BMCs (Figure 3I), which was expected, as direct intraosseous injection results in faster tumor cell proliferation.

**Figure 4:**
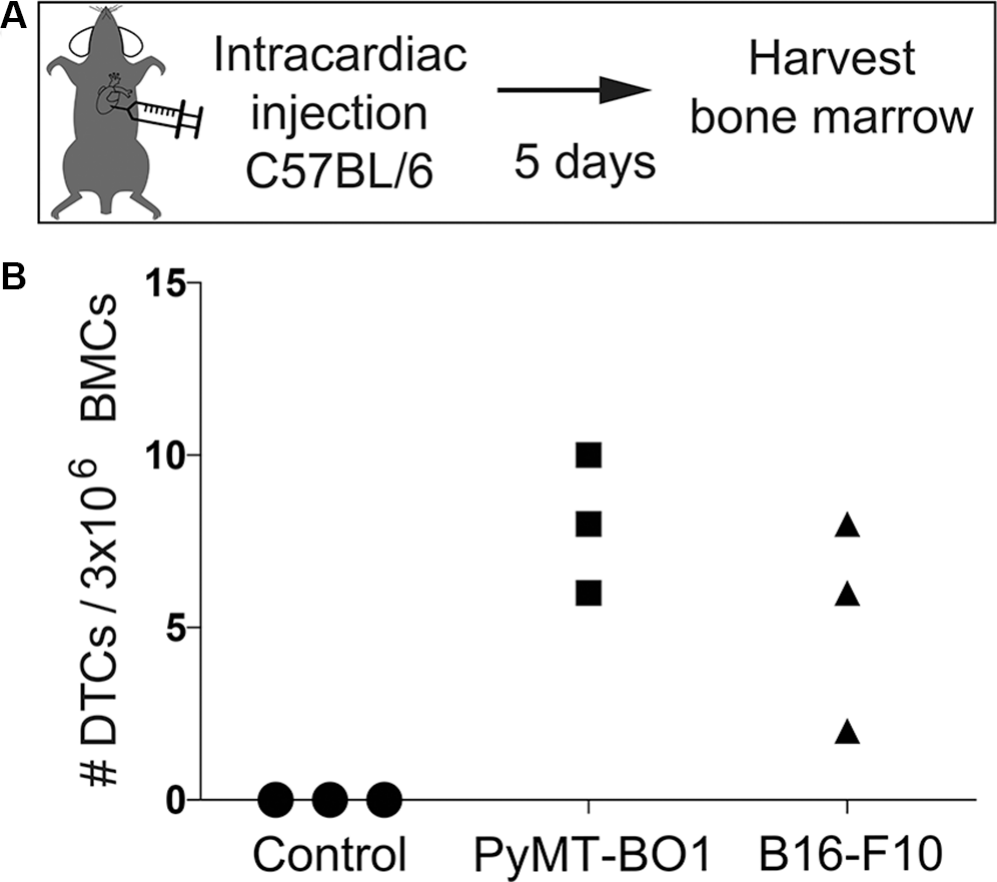
Detection and quantification of murine DTCs in syngeneic murine bone marrow (**A**) Diagram of the intracardiac injection strategy in C57BL/6 mice. (**B**) Quantification of DTCs from bone marrow of C57BL/6 mice following intracardiac injection of murine bone metastatic Polyoma Middle T antigen (PyMT) or bone metastatic B16-F10 melanoma cells.

While xenograft models represent an essential tool in cancer biology research, syngeneic and genetically engineered mouse models represent immune-competent and clinically relevant models of bone metastasis. Therefore, we sought to detect murine tumor cells in murine bone marrow (syngeneic and genetically engineered models). We injected GFP-labeled murine cancer lines PyMT-BO1 (bone metastatic variant of the PyMT breast cancer line) and B16-F10 (bone metastatic variant of the B16 melanoma line) (Supplementary Table S1) IC into C57BL/6 mice, which is the genetic background from which these lines were derived (Figure 4A). We consistently detected bone marrow DTCs in these cells using their endogenous GFP expression (Figure 4B).

**Figure 5:**
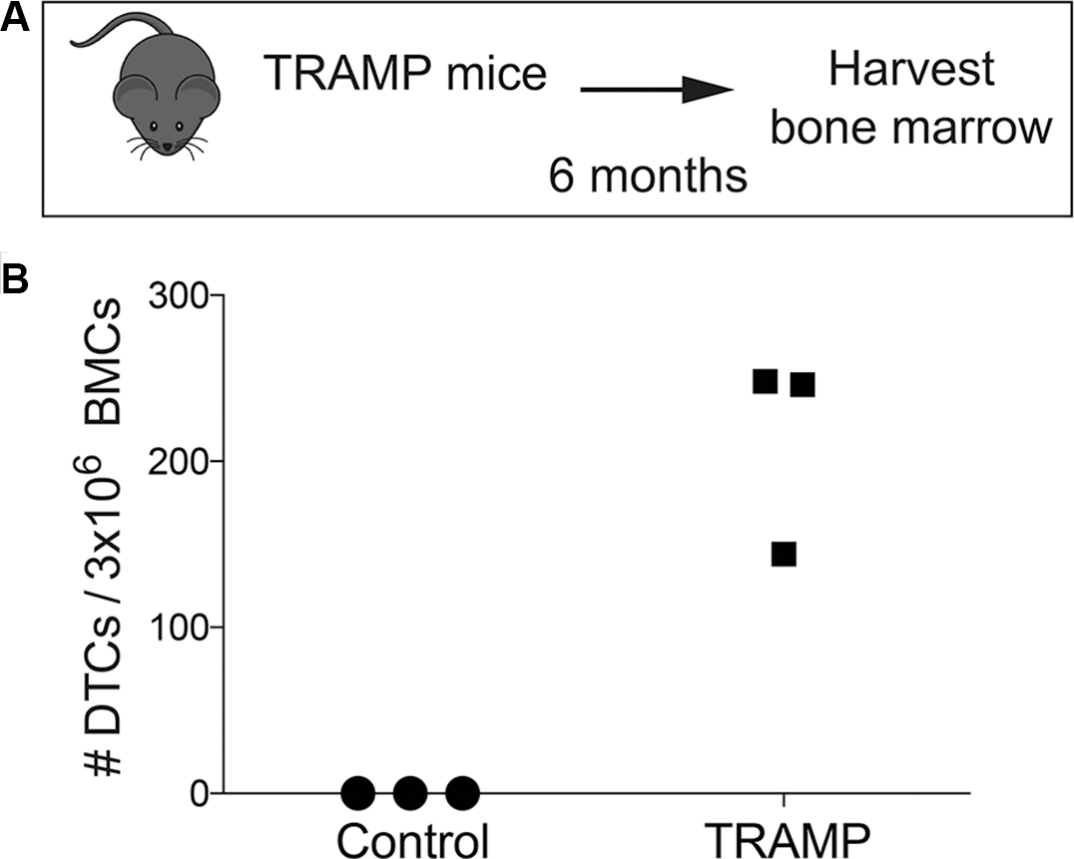
Detection and quantification of murine DTCs in transgenic TRAMP murine bone marrow (**A**) Diagram of the timing strategy of harvesting bone marrow from TRAMP mice. (**B**) Quantification of DTCs from the bone marrow of TRAMP mice with prostate cancer. Tumor cells found in this bone marrow came directly from the primary tumors of these mice.

Notably, we also were able to detect bone marrow DTCs from 6 month old genetically engineered transgenic adenocarcinoma of the mouse prostate (TRAMP) mice (Figure 5A) [25], using an antibody against SV40 T antigen (Figure 5B). Even though these mice showed no evidence of bone metastatic lesions, we found hundreds of bone marrow DTCs (Figure 5B). It is important to note that genetically engineered models most directly recapitulate the entire process of bone metastasis, as any tumor cells detected in these mice came directly from a spontaneous primary prostate tumor and disseminated to the bone marrow.

**Figure 6:**
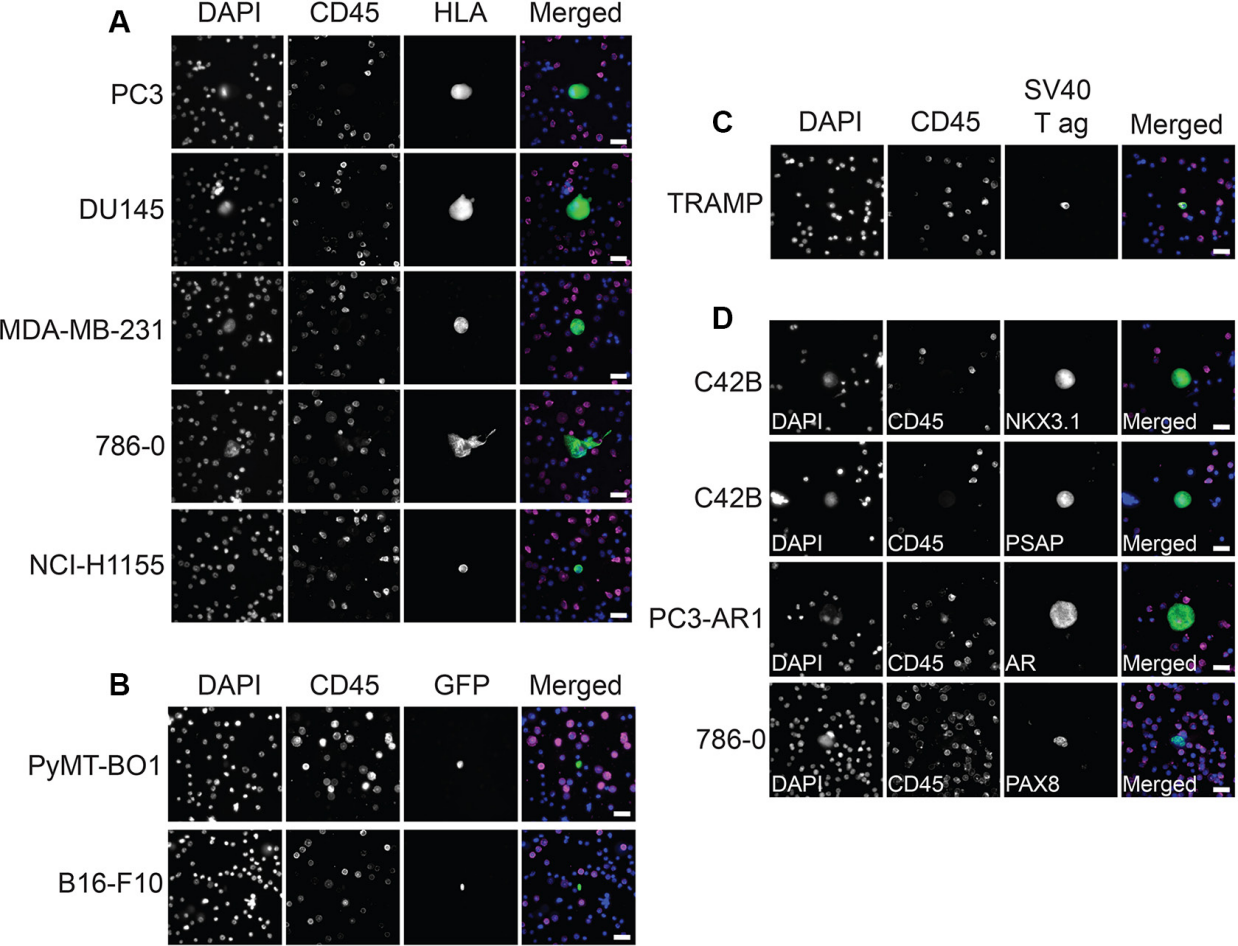
Imaging of bone marrow DTCs and disease-specific markers (**A**) Representative fluorescent images of human DTCs found in murine bone marrow following intracardiac injection. DAPI stains nuclei, CD45 stains BMCs, and HLA stains DTCs. (**B**) Representative fluorescent images of murine DTCs found in murine bone marrow following syngeneic intracardiac injection of GFP-labeled cells. (**C**) Representative fluorescent images of SV40 T antigen-positive murine DTCs found in transgenic TRAMP murine bone marrow. (**D**) Representative fluorescent images of cancer cells spiked into murine bone marrow, stained with tissue-specific markers. NKX3.1, prostate-specific acid phosphatase (PSAP), and androgen receptor (AR) are prostate-specific proteins and stain prostate cell lines C42B and PC3-AR1. PAX8 is a kidney-specific protein and stains kidney cell line 786-0. Scale bars = 20 μm.

**Figure 7:**
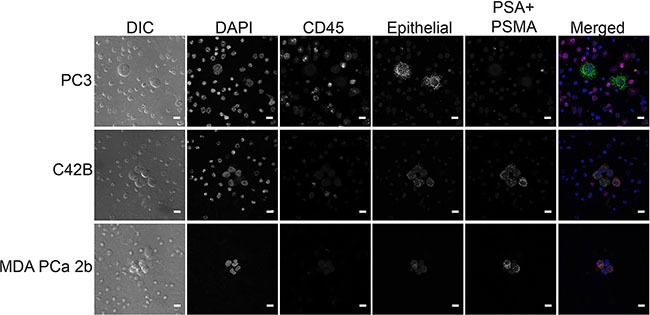
RNA *in situ* hybridization staining in prostate cancer cell lines Representative fluorescent images of DTCs found in murine bone marrow using RISH. DIC (differential interference contrast) shows cells without fluorescence, DAPI stains nuclei, and CD45 stains BMCs. The prostate cocktail detects prostate-specific antigen (PSA, gene name *KLK3*) and prostate-specific membrane antigen (PSMA, gene name *FOLH1*). The epithelial cocktail detects epithelial cell adhesion molecule (EpCAM, gene name *EPCAM*) and cytokeratins 8, 18, and 19 (gene names *KRT8*, *KRT18*, and *KRT19*). Scale bars = 10 μm.

This protocol allows for high resolution imaging of detected DTCs (Figure 6A–6C), and multiple antibodies can be added to allow for further biological investigation. For example, tissue-specific markers such as NKX3.1, prostate-specific acid phosphatase (PSAP), and androgen receptor (AR) (prostate cancer markers) or PAX8 (kidney cancer marker) could be used to confirm the origin of the detected tumor cells (Figure 6D). Fluorescent RNA *in situ* hybridization (RISH) can also be used in this protocol in place of or in combination with immunofluorescence (Figure 7). We used cocktails of RNA probes to detect mRNA in DTCs (RNAScope Multiplex Fluorescent Assay, Advanced Cell Diagnostics). The prostate cocktail detects prostate-specific antigen (PSA, gene name *KLK3*) and prostate-specific membrane antigen (PSMA, gene name *FOLH1*). The epithelial cocktail detects epithelial cell adhesion molecule (EpCAM, gene name *EPCAM*) and cytokeratins 8, 18, and 19 (gene names *KRT8*, *KRT18*, and *KRT19*). C42B and MDA PCa 2b cells express epithelial markers as well as PSA and PSMA, whereas PC3 cells do not express PSA or PSMA but do express epithelial markers. This expression pattern was apparent in our samples (Figure 7). RISH allows for the detection of various RNAs, from mRNA to non-coding RNA.

## DISCUSSION

We have developed a novel method to detect and quantify DTCs in murine bone marrow. We have found human DTCs in immunodeficient NSG mice consistently across five different bone-tropic cancer lines, derived from prostate, breast, lung, and kidney cancer tissue. We further show that tissue-specific markers (PSAP, NKX3.1, AR, and PAX8) and RISH (PSMA and PSA) can also be used to detect and characterize DTCs. We also demonstrate detection of mouse DTCs in immune-competent syngeneic models using endogenous GFP expression in two different cancer lines. Finally, we have found that even native tumor cells in genetically engineered mice can be found in the bone marrow of non-metastatic mice, as we found prostate cancer DTCs in the bone marrow of TRAMP mice.

These findings demonstrate that this protocol can be used for xenograft, syngeneic, and transgenic mouse models, and provides the flexibility to be used for multiple purposes. Human DTCs can be detected using HLA, and this staining can be coupled with a myriad of other antibodies to study various biological processes or expression levels of protein. For mouse-in-mouse models, tumor cells can be detected using endogenous fluorescent proteins, reporter models (such as the mT/mG model [26, 27]), model-specific markers (such as SV40 T antigen), or tissue-specific markers (such as NKX3.1 for prostate tumor cells).

This protocol can visualize a representative population of DTCs without removing tumor cells based on positive or negative selection. It represents an unbiased way to detect all tumor cells present in a sample. This protocol is also extremely sensitive, as it can detect single DTCs from a sample containing millions of BMCs. This is especially important in the study of certain DTC-specific biological processes, such as dormancy, that requires detection of single cells, rather than detection of metastatic tumors. In summary, we present a relatively simple, selection-free method for bone marrow DTC detection that can be used in a myriad of experimental mouse models for the study of DTCs and bone metastasis.

## MATERIALS AND METHODS

### Cell culture

Cells were cultured at 37°C and 5% CO_2_. Cell lines PC3 [28], DU145 [29], C42B [30], bone metastatic MDA-MB-231 [31], and 786–0 [32] cells were grown in RPMI media (Life Technologies, Grand Island, NY), 10% FBS. VCaP [33] cells were grown in DMEM Glutamax media (Life Technologies, Grand Island, NY), 10% FBS. NCI-H1155 [34] cells were grown in RPMI media, 5% FBS. PC3-AR1 cells were grown in F12K media containing 10% charcoal stripped serum and 2 g/mL puromycin, as previously described [35, 36]. The PyMT-BO1 cell line is a bone metastatic variant of the PyMT cell line (originally provided by Dr. DeNardo, Washington University, St. Louis) which was isolated from a fully invasive mammary tumor that spontaneously arose at day 120 in a C57BL/6 background MMTV-PyMT mouse [37]. PyMT-BO1 cells were grown in DMEM media, 10% FBS. B16-F10 [38] cells were grown in DMEM media, 10% FBS, and 1 mg/mL G418 (Corning, Manassas, VA). MDA PCa 2b cells [39] (American Type Culture Collection, Manassas, VA) were grown in BRFF-HPC1 medium (Athena ES, Baltimore, MD), 20% FBS, 50 μg/mL gentamicin (Thermo Fisher, Waltham, MA).

### Mouse models

The Johns Hopkins Institutional Animal Care and Use Committee approved all experiments involving mice. Human cells were injected into immunodeficient NSG mice. Murine cells were injected into immune-competent C57BL/6 mice (Jackson Labs, Bar Harbor, ME). For intracardiac injections, 1 million cancer cells in DPBS were injected into the left ventricle of the heart while the mouse was anesthetized using 2–3% inhaled isoflurane. For subcutaneous injections, 200,000 cancer cells in a 1:1 ratio of DPBS:Matrigel (Corning, Manassas, VA) were injected in the left posterior flank. For intratibial injections into NSG mice, 200,000 cells in DPBS were injected into the tibial cavity while the mouse was anesthetized using 2–3% inhaled isoflurane. The transgenic adenocarcinoma of the mouse prostate (TRAMP) model has been described previously [25]. Mice were euthanized using CO_2_ asphyxiation. For bone marrow isolation from the tibia and femur, the hind limbs were removed from euthanized mice. The growth plates were exposed, and bone marrow was harvested via centrifugation [24].

### Tumor cell isolation and staining protocol

This technique was built upon a previously described method for isolation of CTCs from human blood [40–43]. Before staining, bone marrow samples were cleared of red blood cells using RBC lysis buffer (Roche, Mannheim, Germany). For RISH, RBCs were separated from BMCs and tumor cells via centrifugation in isotonic Percoll (GE Healthcare, Uppsala, Sweden) prepared at a density of 1.102 g/mL. Cells were placed onto adhesion slides (product number 0906000, Paul Marienfeld GmbH & Co. KG, Lauda-Königshofen, Germany) at a concentration of 3 million BMCs per slide. Cells were fixed using 4% paraformaldehyde. Cells were permeabilized using 100% methanol; however for samples containing DTCs with endogenous GFP expression, permeabilization was performed using Triton X-100. Blocking was done with 10% goat serum (Life Technologies, Grand Island, NY) plus mouse TruStain fcX (BioLegend, San Diego, CA, 1:100). Cells were stained with a variety of antibodies: HLA-A (EP1395Y, Abcam, Cambridge, MA, 1:100), CD45 (30-F11, BioLegend, San Diego, CA, 1:100), SV40 T antigen (PAb416, Abcam, Cambridge, MA, 1:100), PAX8 (EPR18715, Abcam, Cambridge, MA, 1:100), NKX3.1 (Athena 0314, Baltimore, MD, 1:200), PSAP (D3Y5P, Cell Signaling, Danvers, MA, 1:200), anti-rabbit-488 (Life Technologies, Grand Island, NY A11034, 1:1000), anti-rat-647 (Life Technologies, Grand Island, NY A21247, 1:1000), anti-mouse-488 (Life Technologies, Grand Island, NY A21121, 1:1000). Cells were also stained using RNA probe cocktails, as described (RNAScope Multiplex Fluorescent Assay, Advanced Cell Diagnostics, Hayward, CA). Diamond anti-fade mounting media containing DAPI (Life Technologies, Grand Island, NY) was used to coverslip the slides.

### Scanning, quantification, and imaging

After staining, slides were imaged and scanned using a Zeiss Axio Imager Z2 combined with Metasystems (Newton, MA) Metafer5 – MetaCyte scanning software. During the scanning procedure, galleries of cells were generated by the software based on fluorescence intensity. These galleries were manually reviewed by trained personnel, and true DTCs were chosen based on the following criteria: DAPI+HLA+CD45−, and the cells needed to have cellular shape and morphology and have consistent staining pattern. The software did not always recognize clusters or aggregates of DTCs, so during manual review, the nuclei of cellular clusters were counted, and the total number of DTCs in that sample was updated with the cells from clusters. Further imaging was performed on Zeiss Observer Z1 and confocal Zeiss LSM780 microscopes using the ZEN software package.

## SUPPLEMENTARY MATERIALS


