# Supplementary material for: A simple selection-free method for detecting disseminated tumor cells (DTCs) in murine bone marrow
Source: Oncotarget. 2016 Sep 13;7(43):69794–803. doi: 10.18632/oncotarget.12000 (PMC5342516; doi:10.18632/oncotarget.12000)
Supplement: Supplementary file 1 [file oncotarget-07-69794-s001.pdf]

A simple selection-free method for detecting disseminated tumor cells (DTCs) in murine bone marrow

Supplementary Materials

Supplementary Table S1: List of all cancer cell lines used in this paper

Cell Line	Cancer Type	Origin	Reference(s)
PC3	Prostate	Human bone metastasis	28
DU145	Prostate	Human dural metastasis	29
C42B	Prostate	Human lymph node metastasis	30
VCaP	Prostate	Human bone metastasis	33
MDA PCa 2b	Prostate	Human bone metastasis	39
PC3-AR1	Prostate	Human bone metastasis; AR-expressing variant	35,36
MDA-MB-231	Breast	Human pleural effusion; bone metastatic variant	31
786-0	Kidney	Human kidney tumor	32
NCI-H1155	Lung	Human lymph node metastasis	34
PyMT-BO1-GFP	Breast	Murine tumor; bone metastatic variant	37
B16-F10-GFP	Melanoma	Murine tumor; bone metastatic variant	38
